# Crystal structure of a Cu^II^ complex with a bridging ligand: poly[[penta­kis­[μ_2_-1,1′-(butane-1,4-di­yl)bis­(1*H*-imidazole)-κ^2^ *N* ^3^:*N* ^3′^]dicopper(II)] tetranitrate tetra­hydrate]

**DOI:** 10.1107/S1600536814024477

**Published:** 2014-11-15

**Authors:** Fayuan Wu, Mengxiang Shang, Shihua Li, Yu Zhao

**Affiliations:** a Heilongjiang Agricultural Vocational and Technical College, JiaMuSi 154007 Heilongjiang, People’s Republic of China

**Keywords:** crystal structure, Cu^II^ complex, 1,1′-(1,4-butane-1,4-di­yl)bis­(1*H*-imidazole), three-dimensional coordination polymer

## Abstract

A novel two-dimensional→three-dimensional Cu^II^ coordination polymer, {[Cu_2_(C_10_H_14_N_4_)_5_](NO_3_)_4_·4H_2_O}_*n*_, based on the 1,1′-(butane-1,4-di­yl)bis­(1*H*-imidazole) (biim) ligand and containing one crystallographically unique Cu^II^ atom, has been synthesized under hydro­thermal conditions. The Cu^II^ atom is coordinated by five N atoms from biim ligands, one of which has crystallographically imposed inversion symmetry, giving rise to a slightly distorted CuN_5_ square-pyramidal geometry. The Cu^II^ cations are linked by biim ligands to give a 4^4^ layer; the layers are further bridged by biim ligands, generating a double sheet with a thickness of 14.61 Å. The sheet features rhombic Cu_4_(biim)_4_ windows built up from four Cu^II^ centers and four biim ligands with dimensions of 14.11 × 14.07 Å^2^. Each window of a layer is penetrated directly by the biim ligand of the adjacent net, giving a two-dimensional→three-dimensional entangled framework.

## Abstract 2

A novel two-dimensional→ three-dimensional Cu^II^ coordination polymer based on the 1,1′-(1,4-butane-1,4-di­yl)bis­(1*H*-imidazole) ligand, containing one crystallographically unique Cu^II^ centre has been synthesized under hydro­thermal conditions.

## Chemical context   

In the past decade, entangled systems of metal–organic frameworks (MOFs) have attracted great attention because of their undisputed aesthetic topological structures, fascinating properties and applications, such as mol­ecular machines and sensor devices, and potential biological applications (Carlucci *et al.*, 2003*a* ▶; Bu *et al.*, 2004 ▶; Batten & Robson, 1998 ▶; Perry *et al.*, 2007 ▶; Yang *et al.*, 2012 ▶; Baburin *et al.*, 2005 ▶; Blatov *et al.*, 2004 ▶). Currently, many chemists are making great contrib­utions to this field, and a number of compounds with entangled framework structures have been synthesized and characterized, which are based on N-donor ligands due to their diversity in coord­ination modes and their versatile conformations (Murphy *et al.*, 2005 ▶; Wu *et al.*, 2011*a* ▶; Yang *et al.*, 2008 ▶; Zhang *et al.*, 2013 ▶). However, the controlled synthesis of crystals with entangled framework structures is still a significant challenge, although many entangled coordination compounds of this sort have already been obtained (Carlucci *et al.*, 2003*b* ▶; Batten, 2001 ▶; Wu *et al.*, 2011*b* ▶). According to previous literature, the construction of MOFs mainly depends on the nature of the organic ligands, metal ions, the temperature, the pH value, and so on (James, 2003 ▶; Chen *et al.*, 2010 ▶; Ma *et al.*, 2004 ▶).

Recently, 1,1′-(1,4-butanedi­yl)bis­(imidazole) and carboxyl­ate ligands have frequently been employed in the construction of coordination compounds due to their flexible character, and coordination compounds displaying different structural motifs have been reported (Wen *et al.*, 2005 ▶; Chen *et al.*, 2009 ▶; Dong *et al.*, 2007 ▶). However, the syntheses of complexes based on inorganic ions have been scarcely been reported.

 It is inter­esting to note that the Cu^II^ complexes based on inorganic counter-ions and the biim ligand, [Cu(biim)_2_(H_2_O)]Cl_2_·5H_2_O (II), [Cu(biim)_2_(H_2_O)](NO_3_)_2_·H_2_O (III) and [Cu(biim)_2_]SO_4_·8H_2_O (IV), were synthesized at room temperature (Ma *et al.*, 2004 ▶). In (II), (III) and (IV), the Cu^II^ cations are bridged by biim ligands, forming infinite 4^4^ networks that contain 44-membered rings. It is worth mentioning that no inter­penetration occurs in (II) and (III), while in (IV), two 4^4^ networks are inter­penetrated in a parallel fashion, forming a two-dimensional →two-dimensional sheet. In the present work, we describe the synthesis and structure of one such entangled Cu^II^ complex, the title compound (I), [Cu_2_(C_10_H_14_N_4_)_5_](NO_3_)_4_·4H_2_O, which exhibits a novel two-dimensional→three-dimensional polymeric structure, and which was prepared under hydro­thermal conditions instead of at room temperature. 
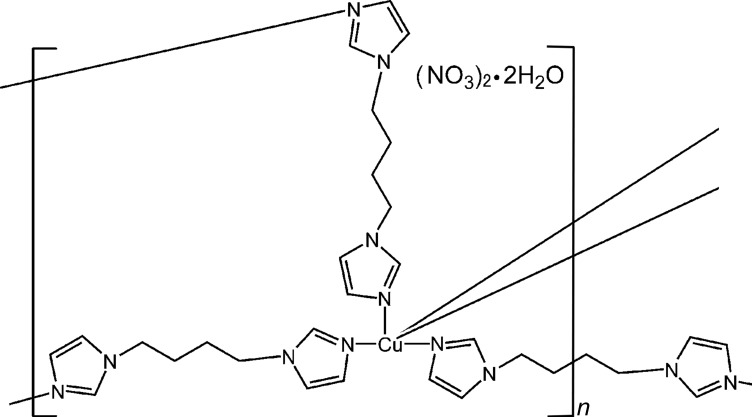


## Structural commentary   

**Figure 1:**
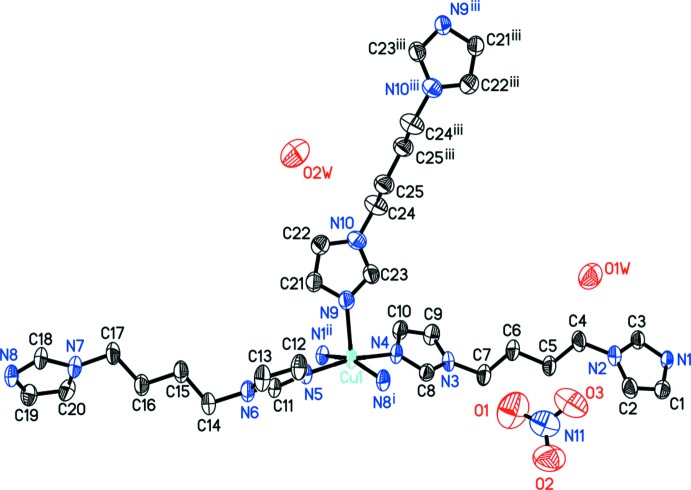
The molecular entities in the structure of the title compound, with anisotropic displacement ellipsoids drawn at the 30% probability level. H atoms are omitted for clarity. [Symmetry codes: (i) *x*, −*y* + 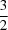, *z* − 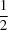; (ii) *x*, −*y* + 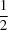, *z* + 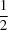; (iii) −*x* + 1, −*y* + 1, −*z* + 1.]

The structure of compound, (I) (Fig. 1 ▶), contains one Cu^II^, two and one half biim ligands, two nitrate ions and two water mol­ecules per asymmetric unit. The Cu^II^ cation is five-coordinated and exhibits a distorted CuN_5_ square-pyramidal coordination geometry from the biim ligands (Table 1 ▶). The *cis* basal N—Cu—N bond angles range from 88.42 (15) to 90.72 (15)°, and the apical bond angles from 92.02 (14) to 101.23 (15)°. 

## Topological features   

**Figure 2:**
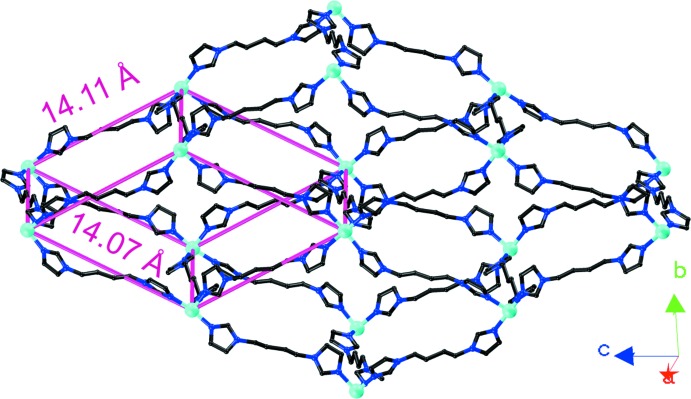
The two-dimensional double layer with large windows in (I).

**Figure 3:**
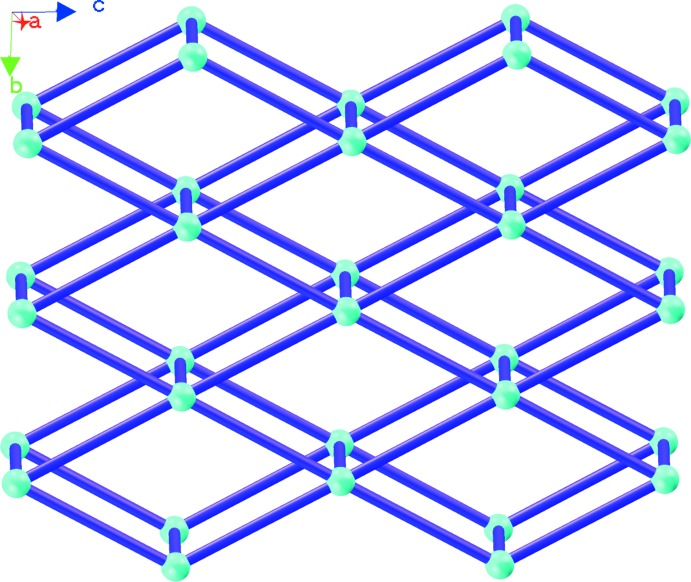
The topology of the two-dimensional layer in (I).

The Cu^II^ cations are linked by biim ligands, giving a 4^4^ layer; the layers are further bridged by biim ligands at nearly vertical directions, generating a double sheet with a thickness of 14.61 Å (Fig. 2 ▶). The sheet exhibits Cu_4_(biim)_4_ windows built up from four Cu^II^ atoms and four biim ligands with dimensions of 14.11 × 14.07 Å^2^. From a topological viewpoint, the sheet reveals a 5-connected topology, in which the Cu atom acts as a 5-connected node and the biim ligand is regarded as a linker. Considering the composition, the Schläfli symbol of the two-dimensional network can be defined as 4^8^.6^2^ (Fig. 3 ▶).

**Figure 4:**
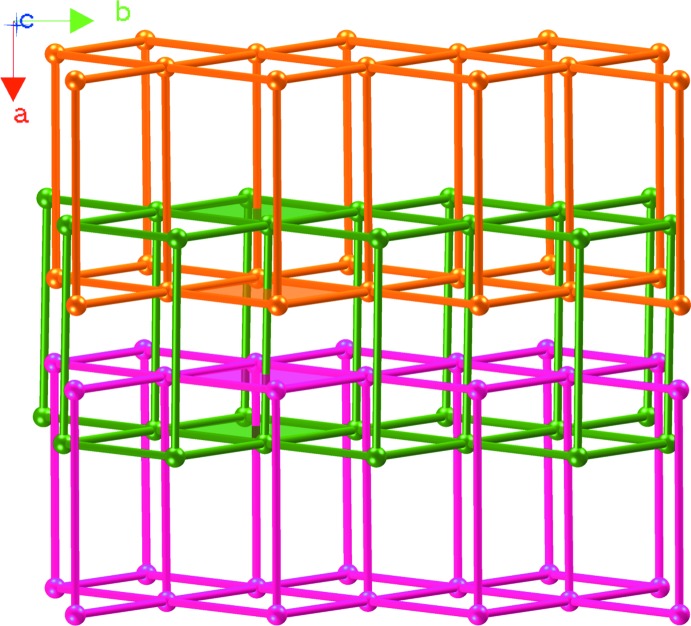
The two-dimensional→three-dimensional framework in (I).

It is noteworthy that every Cu_4_(biim)_4_ unit of each layer is threaded through simultaneously by the biim ligand from an adjacent layer in a parallel fashion, forming a two-dimen­sional→three-dimensional entangled framework, as highlighted in Fig. 4 ▶. All sheets are identical, and all the Cu_4_(biim)_4_ windows are equivalent. As far as we know, so far only a few examples of two-dimensional→three-dimensional entangled structures have been observed: the networks in these are mainly focused on 4^4^ and 6^3^ topologies. Two-dimensional→three-dimensional entangled frameworks with 4^8^.6^2^ topology have scarcely been reported.

It should be pointed out that although the starting materials used for syntheses of (I) and the related compound (III) are the same, their complex structures are entirely different (Ma *et al.*, 2004 ▶). The structure of (III) can be symbolized as a 4^4^ net, and has no inter­penetration. Although it is hard to propose definitive reasons as to why compounds (I) and (III) adopt different configurations, it can be speculated that pH values and temperature may exert an important influence on the resulting architectures.

## Synthesis and crystallization   

A mixture of biim (0.057 g, 0.3 mmol), Cu(NO_3_)_2_·3H_2_O (0.048 g, 0.2 mmol) and water (15 ml) was mixed and stirred at room temperature for 10 min. The mixture was adjusted with 1 *M* HNO_3_ to pH ≃ 5 and then sealed in a 25 ml Teflon-lined autoclave and heated at 443 K for three days. Then the mixture was cooled to room temperature, and black–blue crystals of (I) were obtained in 56% yield based on Cu^II^. Elemental analysis, found: C 42.85, N 24.14, H 5.56%; calculated for C_25_H_39_CuN_12_O_8_ (*M*_r_ = 699.22): C 42.94, N 24.04, H 5.62%.

## Refinement   

Crystal data, data collection and structure refinement details are summarized in Table 2 ▶. All H atoms bonded to C atoms were positioned geometrically and refined as riding atoms,with C—H distances of 0.93 (aromatic) or 0.96 Å (CH_2_) with *U*_iso_(H) = 1.2*U*_eq_(C). H atoms bonded to O atoms were located from difference maps, refined with O—H = 0.84 (1) and H⋯H = 1.40 (1) Å and with *U*_iso_(H) = 1.5*U*_eq_(O). One NO_3_ group was highly disordered and could not be modelled successfully (geometries, adp’s). After using the SQUEEZE (Spek, 2014 ▶) routine of *PLATON* (Spek, 2009 ▶), refinement converged smoothly.

## Supplementary Material

Crystal structure: contains datablock(s) I, New_Global_Publ_Block. DOI: 10.1107/S1600536814024477/fk2083sup1.cif

Structure factors: contains datablock(s) I. DOI: 10.1107/S1600536814024477/fk2083Isup2.hkl

CCDC reference: 1033141

Additional supporting information:  crystallographic information; 3D view; checkCIF report

## Figures and Tables

**Table 1:** Selected geometric parameters (, )

Cu1N1^i^	2.012(4)	Cu1N4	2.043(4)
Cu1N8^ii^	2.013(4)	Cu1N9	2.220(4)
Cu1N5	2.019(4)		
			
N1^i^Cu1N8^ii^	161.25(15)	N5Cu1N4	170.05(15)
N1^i^Cu1N5	90.72(15)	N1^i^Cu1N9	97.52(15)
N8^ii^Cu1N5	88.42(15)	N8^ii^Cu1N9	101.23(15)
N1^i^Cu1N4	88.91(15)	N5Cu1N9	92.02(14)
N8^ii^Cu1N4	88.74(15)	N4Cu1N9	97.89(15)

Symmetry codes: (i) 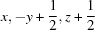; (ii) 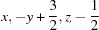.

**Table 2:** Experimental details

Crystal data	
Chemical formula	[Cu_2_(C_10_H_14_N_4_)_5_](NO_3_)_4_4H_2_O
*M*_r_	1398.44
Crystal system, space group	Orthorhombic, *P* *b* *c* *a*
Temperature (K)	293
*a*, *b*, *c* ()	20.034(4), 13.057(3), 24.979(5)
*V* (^3^)	6534(2)
*Z*	4
Radiation type	Mo *K*
(mm^1^)	0.73
Crystal size (mm)	0.21 0.17 0.14

Data collection	
Diffractometer	Oxford Diffraction Gemini R Ultra
Absorption correction	Multi-scan (*CrysAlis PRO*; Agilent, 2012 ▶)
*T*_min_, *T*_max_	0.859, 0.911
No. of measured, independent and observed [*I* > 2(*I*)] reflections	48000, 5763, 3398
*R*_int_	0.111
(sin /)_max_ (^1^)	0.595

Refinement	
*R*[*F* ^2^ > 2(*F* ^2^)], *wR*(*F* ^2^), *S*	0.060, 0.172, 1.03
No. of reflections	5763
No. of parameters	391
No. of restraints	4
H-atom treatment	H atoms treated by a mixture of independent and constrained refinement
_max_, _min_ (e ^3^)	0.34, 0.39

Computer programs: *CrysAlis PRO* (Agilent, 2012 ▶), *SHELXS97*, *SHELXL97* and *SHELXTL* (Sheldrick, 2008 ▶).

## supplementary crystallographic information

### Crystal data

**Table:**

[Cu_2_(C_10_H_14_N_4_)_5_](NO_3_)_4_·4H_2_O	*F*(000) = 2928
*M**_r_* = 1398.44	*D*_x_ = 1.422 Mg m^−^^3^
Orthorhombic, *P**b**c**a*	Mo *K*α radiation, λ = 0.71073 Å
Hall symbol: -P 2ac 2ab	θ = 3.0–25°
*a* = 20.034 (4) Å	µ = 0.73 mm^−^^1^
*b* = 13.057 (3) Å	*T* = 293 K
*c* = 24.979 (5) Å	Block, blue
*V* = 6534 (2) Å^3^	0.21 × 0.17 × 0.14 mm
*Z* = 4	

### Data collection

**Table:**

Oxford Diffraction Gemini R Ultra diffractometer	5763 independent reflections
Radiation source: fine-focus sealed tube	3398 reflections with *I* > 2σ(*I*)
Graphite monochromator	*R*_int_ = 0.111
Detector resolution: 10.0 pixels mm^-1^	θ_max_ = 25.0°, θ_min_ = 3.0°
ω scan	*h* = −23→23
Absorption correction: multi-scan (*CrysAlis PRO*; Agilent, 2012)	*k* = −15→15
*T*_min_ = 0.859, *T*_max_ = 0.911	*l* = −29→29
48000 measured reflections	

### Refinement

**Table:**

Refinement on *F*^2^	Primary atom site location: structure-invariant direct methods
Least-squares matrix: full	Secondary atom site location: difference Fourier map
*R*[*F*^2^ > 2σ(*F*^2^)] = 0.060	Hydrogen site location: inferred from neighbouring sites
*wR*(*F*^2^) = 0.172	H atoms treated by a mixture of independent and constrained refinement
*S* = 1.03	*w* = 1/[σ^2^(*F*_o_^2^) + (0.0868*P*)^2^] where *P* = (*F*_o_^2^ + 2*F*_c_^2^)/3
5763 reflections	(Δ/σ)_max_ = 0.001
391 parameters	Δρ_max_ = 0.34 e Å^−^^3^
4 restraints	Δρ_min_ = −0.39 e Å^−^^3^

### Special details

**Table:**

Geometry. All s.u.'s (except the s.u. in the dihedral angle between two l.s. planes) are estimated using the full covariance matrix. The cell s.u.'s are taken into account individually in the estimation of s.u.'s in distances, angles and torsion angles; correlations between s.u.'s in cell parameters are only used when they are defined by crystal symmetry. An approximate (isotropic) treatment of cell s.u.'s is used for estimating s.u.'s involving l.s. planes.
Refinement. Refinement of *F*^2^ against ALL reflections. The weighted *R*-factor *wR* and goodness of fit *S* are based on *F*^2^, conventional *R*-factors *R* are based on *F*, with *F* set to zero for negative *F*^2^. The threshold expression of *F*^2^ > 2σ(*F*^2^) is used only for calculating *R*-factors(gt) *etc*. and is not relevant to the choice of reflections for refinement. *R*-factors based on *F*^2^ are statistically about twice as large as those based on *F*, and *R*- factors based on ALL data will be even larger.

### Fractional atomic coordinates and isotropic or equivalent isotropic displacement parameters (Å^2^)

**Table:**

	*x*	*y*	*z*	*U*_iso_*/*U*_eq_	
Cu1	0.85764 (3)	0.50167 (4)	0.55687 (2)	0.03364 (19)	
C1	0.9175 (3)	0.1747 (4)	0.1200 (2)	0.0512 (14)	
H1	0.9487	0.1890	0.0934	0.061*	
C2	0.9112 (3)	0.2278 (4)	0.1654 (2)	0.0526 (14)	
H2	0.9368	0.2834	0.1763	0.063*	
C3	0.8368 (2)	0.1055 (3)	0.16238 (18)	0.0409 (12)	
H3	0.8012	0.0633	0.1717	0.049*	
C4	0.8320 (3)	0.2147 (4)	0.2448 (2)	0.0496 (13)	
H4A	0.7958	0.1689	0.2543	0.060*	
H4B	0.8140	0.2835	0.2422	0.060*	
C5	0.8852 (3)	0.2117 (4)	0.28812 (19)	0.0447 (12)	
H5A	0.9216	0.2571	0.2785	0.054*	
H5B	0.9029	0.1428	0.2911	0.054*	
C6	0.8558 (3)	0.2446 (4)	0.34183 (19)	0.0478 (13)	
H6A	0.8360	0.3120	0.3382	0.057*	
H6B	0.8208	0.1971	0.3521	0.057*	
C7	0.9083 (3)	0.2472 (4)	0.3846 (2)	0.0516 (14)	
H7A	0.9273	0.1792	0.3884	0.062*	
H7B	0.9437	0.2930	0.3735	0.062*	
C8	0.8921 (2)	0.3729 (3)	0.45896 (19)	0.0380 (11)	
H8	0.9178	0.4248	0.4439	0.046*	
C9	0.8414 (3)	0.2265 (4)	0.4708 (2)	0.0478 (13)	
H9	0.8256	0.1602	0.4659	0.057*	
C10	0.8286 (2)	0.2885 (4)	0.5121 (2)	0.0452 (12)	
H10	0.8021	0.2715	0.5414	0.054*	
C11	0.9033 (2)	0.6239 (3)	0.65298 (18)	0.0375 (11)	
H11	0.9263	0.5687	0.6677	0.045*	
C12	0.8446 (3)	0.7176 (4)	0.6003 (2)	0.0508 (14)	
H12	0.8189	0.7388	0.5713	0.061*	
C13	0.8606 (3)	0.7759 (4)	0.6433 (2)	0.0522 (14)	
H13	0.8487	0.8439	0.6490	0.063*	
C14	0.9217 (3)	0.7440 (4)	0.7300 (2)	0.0511 (13)	
H14A	0.9475	0.8067	0.7273	0.061*	
H14B	0.9510	0.6907	0.7433	0.061*	
C15	0.8656 (3)	0.7597 (4)	0.76881 (19)	0.0476 (13)	
H15A	0.8372	0.6995	0.7691	0.057*	
H15B	0.8388	0.8177	0.7576	0.057*	
C16	0.8926 (3)	0.7786 (4)	0.82474 (19)	0.0475 (13)	
H16A	0.9177	0.7192	0.8365	0.057*	
H16B	0.9227	0.8368	0.8241	0.057*	
C17	0.8365 (3)	0.7994 (4)	0.86372 (19)	0.0472 (13)	
H25A	0.8095	0.8558	0.8506	0.057*	
H25B	0.8081	0.7394	0.8663	0.057*	
C18	0.8399 (2)	0.8982 (3)	0.95009 (17)	0.0382 (11)	
H17	0.8033	0.9395	0.9425	0.046*	
C19	0.9223 (3)	0.8289 (4)	0.99014 (19)	0.0454 (12)	
H18	0.9542	0.8132	1.0160	0.054*	
C20	0.9157 (3)	0.7798 (4)	0.9423 (2)	0.0468 (13)	
H19	0.9417	0.7263	0.9294	0.056*	
C21	0.7128 (2)	0.5565 (4)	0.6069 (2)	0.0452 (12)	
H21	0.7321	0.5919	0.6353	0.054*	
C22	0.6471 (3)	0.5383 (4)	0.6015 (2)	0.0475 (12)	
H22	0.6133	0.5582	0.6249	0.057*	
C23	0.7012 (2)	0.4723 (4)	0.5336 (2)	0.0436 (12)	
H20	0.7100	0.4384	0.5016	0.052*	
C24	0.5780 (3)	0.4428 (4)	0.5328 (2)	0.0567 (15)	
H23A	0.5548	0.4057	0.5608	0.068*	
H23B	0.5889	0.3943	0.5047	0.068*	
C25	0.5322 (2)	0.5232 (4)	0.5104 (2)	0.0510 (14)	
H24A	0.5546	0.5591	0.4816	0.061*	
H24B	0.5218	0.5727	0.5382	0.061*	
N1	0.87215 (18)	0.0969 (3)	0.11766 (14)	0.0363 (9)	
N2	0.8596 (2)	0.1840 (3)	0.19254 (15)	0.0426 (10)	
N3	0.8827 (2)	0.2808 (3)	0.43685 (15)	0.0400 (10)	
N4	0.86013 (19)	0.3808 (3)	0.50499 (14)	0.0386 (9)	
N5	0.87252 (18)	0.6217 (3)	0.60642 (14)	0.0368 (9)	
N6	0.8974 (2)	0.7152 (3)	0.67632 (16)	0.0430 (10)	
N7	0.86318 (19)	0.8252 (3)	0.91747 (15)	0.0406 (9)	
N8	0.8757 (2)	0.9042 (3)	0.99470 (15)	0.0402 (9)	
N9	0.74755 (19)	0.5150 (3)	0.56425 (15)	0.0408 (10)	
N10	0.63959 (19)	0.4843 (3)	0.55460 (17)	0.0460 (10)	
N11	0.9485 (4)	0.4798 (4)	0.2707 (3)	0.0834 (19)	
O1	0.9319 (4)	0.4666 (4)	0.3186 (3)	0.139 (3)	
O2	1.0051 (3)	0.5040 (5)	0.2616 (3)	0.121 (2)	
O1W	0.7338 (3)	−0.0160 (5)	0.2453 (2)	0.1075 (18)	
O3	0.9069 (3)	0.4732 (5)	0.2355 (3)	0.129 (2)	
O2W	0.4859 (3)	0.5308 (6)	0.6429 (3)	0.130 (2)	
H1A	0.6939 (16)	0.002 (8)	0.248 (4)	0.195*	
H2A	0.499 (6)	0.534 (10)	0.6748 (17)	0.195*	
H1B	0.754 (4)	−0.021 (8)	0.275 (2)	0.195*	
H2B	0.436 (6)	0.534 (8)	0.629 (4)	0.195*	

### Atomic displacement parameters (Å^2^)

**Table:**

	*U*^11^	*U*^22^	*U*^33^	*U*^12^	*U*^13^	*U*^23^
Cu1	0.0493 (3)	0.0356 (3)	0.0161 (3)	0.0011 (3)	−0.0011 (2)	0.0008 (2)
C1	0.059 (3)	0.062 (3)	0.032 (3)	−0.021 (3)	0.006 (2)	0.001 (3)
C2	0.073 (4)	0.051 (3)	0.034 (3)	−0.024 (3)	−0.001 (3)	−0.005 (2)
C3	0.052 (3)	0.043 (3)	0.028 (3)	−0.007 (2)	−0.001 (2)	−0.005 (2)
C4	0.060 (3)	0.058 (3)	0.031 (3)	0.004 (3)	−0.004 (2)	−0.022 (2)
C5	0.056 (3)	0.050 (3)	0.028 (3)	0.005 (2)	0.000 (2)	−0.013 (2)
C6	0.062 (3)	0.054 (3)	0.028 (3)	0.005 (3)	0.004 (2)	−0.010 (2)
C7	0.063 (3)	0.061 (3)	0.030 (3)	0.010 (3)	0.003 (2)	−0.009 (3)
C8	0.042 (3)	0.039 (3)	0.033 (3)	−0.004 (2)	0.004 (2)	0.003 (2)
C9	0.067 (4)	0.039 (3)	0.037 (3)	−0.004 (2)	0.004 (3)	0.000 (2)
C10	0.058 (3)	0.047 (3)	0.031 (3)	−0.007 (3)	0.008 (2)	−0.003 (2)
C11	0.049 (3)	0.041 (3)	0.023 (3)	0.007 (2)	0.002 (2)	−0.008 (2)
C12	0.082 (4)	0.044 (3)	0.026 (3)	0.008 (3)	−0.005 (3)	0.004 (2)
C13	0.086 (4)	0.036 (3)	0.034 (3)	0.009 (3)	0.003 (3)	−0.007 (2)
C14	0.062 (3)	0.061 (3)	0.030 (3)	−0.003 (3)	−0.002 (2)	−0.018 (3)
C15	0.063 (3)	0.050 (3)	0.029 (3)	0.000 (3)	−0.002 (2)	−0.008 (2)
C16	0.060 (3)	0.057 (3)	0.025 (3)	−0.006 (3)	−0.001 (2)	−0.008 (2)
C17	0.060 (3)	0.059 (3)	0.023 (3)	0.005 (3)	−0.005 (2)	−0.013 (2)
C18	0.055 (3)	0.036 (2)	0.024 (3)	0.009 (2)	−0.001 (2)	−0.007 (2)
C19	0.056 (3)	0.053 (3)	0.027 (3)	0.003 (3)	−0.001 (2)	0.003 (2)
C20	0.054 (3)	0.051 (3)	0.035 (3)	0.015 (3)	0.007 (2)	−0.007 (2)
C21	0.047 (3)	0.056 (3)	0.033 (3)	0.009 (2)	−0.003 (2)	−0.009 (2)
C22	0.052 (3)	0.044 (3)	0.046 (3)	0.007 (2)	0.005 (3)	−0.004 (2)
C23	0.044 (3)	0.052 (3)	0.035 (3)	0.005 (2)	−0.005 (2)	−0.006 (2)
C24	0.051 (3)	0.048 (3)	0.071 (4)	0.001 (3)	−0.018 (3)	0.003 (3)
C25	0.047 (3)	0.054 (3)	0.052 (3)	−0.001 (2)	−0.003 (3)	0.004 (3)
N1	0.051 (2)	0.040 (2)	0.018 (2)	−0.0064 (18)	−0.0005 (17)	−0.0011 (16)
N2	0.058 (3)	0.046 (2)	0.025 (2)	0.001 (2)	−0.001 (2)	−0.0066 (18)
N3	0.051 (2)	0.043 (2)	0.026 (2)	0.0048 (19)	−0.0063 (19)	−0.0080 (18)
N4	0.057 (2)	0.038 (2)	0.021 (2)	0.0000 (19)	0.0043 (18)	−0.0002 (16)
N5	0.052 (2)	0.038 (2)	0.020 (2)	0.0028 (18)	0.0003 (17)	0.0011 (16)
N6	0.058 (3)	0.046 (2)	0.025 (2)	−0.007 (2)	0.0028 (19)	−0.0071 (19)
N7	0.057 (3)	0.042 (2)	0.023 (2)	0.007 (2)	0.0022 (19)	−0.0060 (18)
N8	0.059 (3)	0.039 (2)	0.023 (2)	0.0006 (19)	−0.0003 (19)	−0.0015 (17)
N9	0.050 (2)	0.044 (2)	0.028 (2)	0.0029 (18)	−0.0026 (18)	−0.0014 (18)
N10	0.045 (2)	0.042 (2)	0.051 (3)	0.0019 (19)	−0.006 (2)	0.0033 (19)
N11	0.097 (5)	0.040 (3)	0.113 (6)	−0.006 (3)	−0.007 (5)	0.009 (3)
O1	0.203 (7)	0.086 (4)	0.129 (6)	−0.018 (4)	0.037 (5)	0.018 (4)
O2	0.081 (4)	0.153 (5)	0.131 (6)	−0.001 (4)	−0.011 (4)	0.041 (4)
O1W	0.117 (5)	0.129 (5)	0.076 (4)	−0.022 (4)	0.011 (3)	0.013 (4)
O3	0.101 (5)	0.131 (5)	0.154 (6)	−0.012 (3)	−0.042 (4)	−0.014 (4)
O2W	0.102 (4)	0.177 (6)	0.110 (5)	−0.019 (4)	0.031 (4)	−0.007 (5)

### Geometric parameters (Å, º)

**Table:**

Cu1—N1^i^	2.012 (4)	C14—H14A	0.9700
Cu1—N8^ii^	2.013 (4)	C14—H14B	0.9700
Cu1—N5	2.019 (4)	C15—C16	1.519 (6)
Cu1—N4	2.043 (4)	C15—H15A	0.9700
Cu1—N9	2.220 (4)	C15—H15B	0.9700
C1—C2	1.337 (7)	C16—C17	1.512 (7)
C1—N1	1.364 (6)	C16—H16A	0.9700
C1—H1	0.9300	C16—H16B	0.9700
C2—N2	1.360 (6)	C17—N7	1.484 (6)
C2—H2	0.9300	C17—H25A	0.9700
C3—N1	1.328 (6)	C17—H25B	0.9700
C3—N2	1.353 (6)	C18—N8	1.327 (6)
C3—H3	0.9300	C18—N7	1.338 (5)
C4—N2	1.472 (6)	C18—H17	0.9300
C4—C5	1.519 (7)	C19—N8	1.361 (6)
C4—H4A	0.9700	C19—C20	1.362 (7)
C4—H4B	0.9700	C19—H18	0.9300
C5—C6	1.527 (6)	C20—N7	1.358 (6)
C5—H5A	0.9700	C20—H19	0.9300
C5—H5B	0.9700	C21—C22	1.345 (7)
C6—C7	1.500 (7)	C21—N9	1.384 (6)
C6—H6A	0.9700	C21—H21	0.9300
C6—H6B	0.9700	C22—N10	1.375 (6)
C7—N3	1.469 (6)	C22—H22	0.9300
C7—H7A	0.9700	C23—N9	1.327 (6)
C7—H7B	0.9700	C23—N10	1.351 (6)
C8—N4	1.320 (5)	C23—H20	0.9300
C8—N3	1.337 (6)	C24—N10	1.453 (6)
C8—H8	0.9300	C24—C25	1.503 (7)
C9—C10	1.336 (6)	C24—H23A	0.9700
C9—N3	1.381 (6)	C24—H23B	0.9700
C9—H9	0.9300	C25—C25^iii^	1.518 (9)
C10—N4	1.373 (6)	C25—H24A	0.9700
C10—H10	0.9300	C25—H24B	0.9700
C11—N5	1.317 (5)	N1—Cu1^iv^	2.012 (4)
C11—N6	1.333 (5)	N8—Cu1^v^	2.013 (4)
C11—H11	0.9300	N11—O2	1.200 (8)
C12—C13	1.354 (7)	N11—O3	1.213 (8)
C12—N5	1.380 (6)	N11—O1	1.254 (8)
C12—H12	0.9300	O1W—H1A	0.839 (10)
C13—N6	1.362 (6)	O1W—H1B	0.839 (10)
C13—H13	0.9300	O2W—H2A	0.842 (10)
C14—N6	1.475 (6)	O2W—H2B	1.06 (11)
C14—C15	1.499 (7)		
			
N1^i^—Cu1—N8^ii^	161.25 (15)	C15—C16—H16A	109.5
N1^i^—Cu1—N5	90.72 (15)	C17—C16—H16B	109.5
N8^ii^—Cu1—N5	88.42 (15)	C15—C16—H16B	109.5
N1^i^—Cu1—N4	88.91 (15)	H16A—C16—H16B	108.1
N8^ii^—Cu1—N4	88.74 (15)	N7—C17—C16	110.8 (4)
N5—Cu1—N4	170.05 (15)	N7—C17—H25A	109.5
N1^i^—Cu1—N9	97.52 (15)	C16—C17—H25A	109.5
N8^ii^—Cu1—N9	101.23 (15)	N7—C17—H25B	109.5
N5—Cu1—N9	92.02 (14)	C16—C17—H25B	109.5
N4—Cu1—N9	97.89 (15)	H25A—C17—H25B	108.1
C2—C1—N1	111.0 (4)	N8—C18—N7	111.4 (4)
C2—C1—H1	124.5	N8—C18—H17	124.3
N1—C1—H1	124.5	N7—C18—H17	124.3
C1—C2—N2	106.1 (4)	N8—C19—C20	110.2 (4)
C1—C2—H2	126.9	N8—C19—H18	124.9
N2—C2—H2	126.9	C20—C19—H18	124.9
N1—C3—N2	110.6 (4)	N7—C20—C19	105.7 (4)
N1—C3—H3	124.7	N7—C20—H19	127.1
N2—C3—H3	124.7	C19—C20—H19	127.1
N2—C4—C5	111.2 (4)	C22—C21—N9	110.2 (4)
N2—C4—H4A	109.4	C22—C21—H21	124.9
C5—C4—H4A	109.4	N9—C21—H21	124.9
N2—C4—H4B	109.4	C21—C22—N10	106.5 (4)
C5—C4—H4B	109.4	C21—C22—H22	126.8
H4A—C4—H4B	108.0	N10—C22—H22	126.8
C4—C5—C6	110.4 (4)	N9—C23—N10	111.5 (4)
C4—C5—H5A	109.6	N9—C23—H20	124.3
C6—C5—H5A	109.6	N10—C23—H20	124.3
C4—C5—H5B	109.6	N10—C24—C25	113.4 (4)
C6—C5—H5B	109.6	N10—C24—H23A	108.9
H5A—C5—H5B	108.1	C25—C24—H23A	108.9
C7—C6—C5	111.2 (4)	N10—C24—H23B	108.9
C7—C6—H6A	109.4	C25—C24—H23B	108.9
C5—C6—H6A	109.4	H23A—C24—H23B	107.7
C7—C6—H6B	109.4	C24—C25—C25^iii^	111.6 (5)
C5—C6—H6B	109.4	C24—C25—H24A	109.3
H6A—C6—H6B	108.0	C25^iii^—C25—H24A	109.3
N3—C7—C6	113.3 (4)	C24—C25—H24B	109.3
N3—C7—H7A	108.9	C25^iii^—C25—H24B	109.3
C6—C7—H7A	108.9	H24A—C25—H24B	108.0
N3—C7—H7B	108.9	C3—N1—C1	104.9 (4)
C6—C7—H7B	108.9	C3—N1—Cu1^iv^	127.7 (3)
H7A—C7—H7B	107.7	C1—N1—Cu1^iv^	127.2 (3)
N4—C8—N3	111.2 (4)	C3—N2—C2	107.3 (4)
N4—C8—H8	124.4	C3—N2—C4	124.9 (4)
N3—C8—H8	124.4	C2—N2—C4	127.7 (4)
C10—C9—N3	106.2 (4)	C8—N3—C9	107.0 (4)
C10—C9—H9	126.9	C8—N3—C7	126.0 (4)
N3—C9—H9	126.9	C9—N3—C7	126.9 (4)
C9—C10—N4	110.0 (4)	C8—N4—C10	105.5 (4)
C9—C10—H10	125.0	C8—N4—Cu1	128.7 (3)
N4—C10—H10	125.0	C10—N4—Cu1	125.8 (3)
N5—C11—N6	111.3 (4)	C11—N5—C12	105.6 (4)
N5—C11—H11	124.3	C11—N5—Cu1	128.9 (3)
N6—C11—H11	124.3	C12—N5—Cu1	125.2 (3)
C13—C12—N5	109.1 (4)	C11—N6—C13	107.7 (4)
C13—C12—H12	125.5	C11—N6—C14	126.6 (4)
N5—C12—H12	125.5	C13—N6—C14	125.6 (4)
C12—C13—N6	106.3 (4)	C18—N7—C20	107.6 (4)
C12—C13—H13	126.8	C18—N7—C17	125.9 (4)
N6—C13—H13	126.8	C20—N7—C17	126.5 (4)
N6—C14—C15	112.0 (4)	C18—N8—C19	104.9 (4)
N6—C14—H14A	109.2	C18—N8—Cu1^v^	125.9 (3)
C15—C14—H14A	109.2	C19—N8—Cu1^v^	129.0 (3)
N6—C14—H14B	109.2	C23—N9—C21	104.9 (4)
C15—C14—H14B	109.2	C23—N9—Cu1	127.9 (3)
H14A—C14—H14B	107.9	C21—N9—Cu1	126.5 (3)
C14—C15—C16	110.5 (4)	C23—N10—C22	107.0 (4)
C14—C15—H15A	109.6	C23—N10—C24	125.9 (5)
C16—C15—H15A	109.6	C22—N10—C24	127.1 (4)
C14—C15—H15B	109.6	O2—N11—O3	122.1 (9)
C16—C15—H15B	109.6	O2—N11—O1	117.9 (8)
H15A—C15—H15B	108.1	O3—N11—O1	120.0 (9)
C17—C16—C15	110.9 (4)	H1A—O1W—H1B	113 (2)
C17—C16—H16A	109.5	H2A—O2W—H2B	127 (10)
			
N1—C1—C2—N2	−1.1 (6)	C13—C12—N5—Cu1	174.6 (3)
N2—C4—C5—C6	179.5 (4)	N1^i^—Cu1—N5—C11	20.7 (4)
C4—C5—C6—C7	−177.2 (4)	N8^ii^—Cu1—N5—C11	−140.6 (4)
C5—C6—C7—N3	178.6 (4)	N4—Cu1—N5—C11	−67.1 (10)
N3—C9—C10—N4	0.6 (6)	N9—Cu1—N5—C11	118.3 (4)
N5—C12—C13—N6	−1.1 (6)	N1^i^—Cu1—N5—C12	−151.1 (4)
N6—C14—C15—C16	174.3 (4)	N8^ii^—Cu1—N5—C12	47.6 (4)
C14—C15—C16—C17	177.3 (4)	N4—Cu1—N5—C12	121.1 (8)
C15—C16—C17—N7	−176.0 (4)	N9—Cu1—N5—C12	−53.6 (4)
N8—C19—C20—N7	−0.9 (6)	N5—C11—N6—C13	0.1 (6)
N9—C21—C22—N10	0.0 (6)	N5—C11—N6—C14	176.0 (4)
N10—C24—C25—C25^iii^	−178.5 (6)	C12—C13—N6—C11	0.6 (6)
N2—C3—N1—C1	−1.4 (5)	C12—C13—N6—C14	−175.3 (4)
N2—C3—N1—Cu1^iv^	−176.9 (3)	C15—C14—N6—C11	−109.5 (6)
C2—C1—N1—C3	1.5 (6)	C15—C14—N6—C13	65.6 (6)
C2—C1—N1—Cu1^iv^	177.1 (4)	N8—C18—N7—C20	1.3 (5)
N1—C3—N2—C2	0.7 (5)	N8—C18—N7—C17	−178.3 (4)
N1—C3—N2—C4	−179.7 (4)	C19—C20—N7—C18	−0.2 (5)
C1—C2—N2—C3	0.2 (6)	C19—C20—N7—C17	179.4 (4)
C1—C2—N2—C4	−179.3 (5)	C16—C17—N7—C18	138.9 (5)
C5—C4—N2—C3	122.3 (5)	C16—C17—N7—C20	−40.6 (7)
C5—C4—N2—C2	−58.3 (7)	N7—C18—N8—C19	−1.8 (5)
N4—C8—N3—C9	0.7 (5)	N7—C18—N8—Cu1^v^	−177.4 (3)
N4—C8—N3—C7	177.0 (4)	C20—C19—N8—C18	1.7 (5)
C10—C9—N3—C8	−0.8 (5)	C20—C19—N8—Cu1^v^	177.1 (3)
C10—C9—N3—C7	−177.1 (4)	N10—C23—N9—C21	−0.2 (5)
C6—C7—N3—C8	−104.1 (6)	N10—C23—N9—Cu1	170.6 (3)
C6—C7—N3—C9	71.6 (6)	C22—C21—N9—C23	0.2 (6)
N3—C8—N4—C10	−0.3 (5)	C22—C21—N9—Cu1	−170.9 (3)
N3—C8—N4—Cu1	178.7 (3)	N1^i^—Cu1—N9—C23	−103.9 (4)
C9—C10—N4—C8	−0.2 (6)	N8^ii^—Cu1—N9—C23	76.3 (4)
C9—C10—N4—Cu1	−179.3 (3)	N5—Cu1—N9—C23	165.1 (4)
N1^i^—Cu1—N4—C8	−134.7 (4)	N4—Cu1—N9—C23	−14.0 (4)
N8^ii^—Cu1—N4—C8	26.7 (4)	N1^i^—Cu1—N9—C21	65.1 (4)
N5—Cu1—N4—C8	−46.8 (11)	N8^ii^—Cu1—N9—C21	−114.7 (4)
N9—Cu1—N4—C8	127.8 (4)	N5—Cu1—N9—C21	−25.9 (4)
N1^i^—Cu1—N4—C10	44.1 (4)	N4—Cu1—N9—C21	155.0 (4)
N8^ii^—Cu1—N4—C10	−154.5 (4)	N9—C23—N10—C22	0.2 (6)
N5—Cu1—N4—C10	132.1 (8)	N9—C23—N10—C24	−176.7 (4)
N9—Cu1—N4—C10	−53.3 (4)	C21—C22—N10—C23	−0.1 (5)
N6—C11—N5—C12	−0.8 (5)	C21—C22—N10—C24	176.8 (4)
N6—C11—N5—Cu1	−173.9 (3)	C25—C24—N10—C23	−110.7 (6)
C13—C12—N5—C11	1.2 (6)	C25—C24—N10—C22	73.0 (7)

Symmetry codes: (i) *x*, −*y*+1/2, *z*+1/2; (ii) *x*, −*y*+3/2, *z*−1/2; (iii) −*x*+1, −*y*+1, −*z*+1; (iv) *x*, −*y*+1/2, *z*−1/2; (v) *x*, −*y*+3/2, *z*+1/2.
